# QTL Detection and Elite Alleles Mining for Stigma Traits in *Oryza sativa* by Association Mapping

**DOI:** 10.3389/fpls.2016.01188

**Published:** 2016-08-09

**Authors:** Xiaojing Dang, Erbao Liu, Yinfeng Liang, Qiangming Liu, Caleb M. Breria, Delin Hong

**Affiliations:** ^1^State Key Laboratory of Crop Genetics and Germplasm Enhancement, College of Agriculture, Nanjing Agricultural UniversityNanjing, China; ^2^Rice Research Institute, Chongqing Academy of Agricultural SciencesChongqing, China

**Keywords:** QTL detection, elite allele, genome-wide association mapping, *Oryza sativa*, stigma traits

## Abstract

Stigma traits are very important for hybrid seed production in *Oryza sativa*, which is a self-pollinated crop; however, the genetic mechanism controlling the traits is poorly understood. In this study, we investigated the phenotypic data of 227 accessions across 2 years and assessed their genotypic variation with 249 simple sequence repeat (SSR) markers. By combining phenotypic and genotypic data, a genome-wide association (GWA) map was generated. Large phenotypic variations in stigma length (STL), stigma brush-shaped part length (SBPL) and stigma non-brush-shaped part length (SNBPL) were found. Significant positive correlations were identified among stigma traits. In total, 2072 alleles were detected among 227 accessions, with an average of 8.3 alleles per SSR locus. GWA mapping detected 6 quantitative trait loci (QTLs) for the STL, 2 QTLs for the SBPL and 7 QTLs for the SNBPL. Eleven, 5, and 12 elite alleles were found for the STL, SBPL, and SNBPL, respectively. Optimal cross designs were predicted for improving the target traits. The detected genetic variation in stigma traits and QTLs provides helpful information for cloning candidate STL genes and breeding rice cultivars with longer STLs in the future.

## Introduction

Cultivated Asian rice (*Oryza sativa* L.) is one of the oldest domesticated crop species in the world and feeds more than one half of the world's population. This widespread utility is attributed to the large geographical range of *O. sativa*, which extends from 43°S (Australia) to 54°N (Mo River, North China) and from 7°E (Italy) to 117°W (California, USA). Two groups of genetically divergent subspecies, *Oryza sativa* subspecies *indica* and *Oryza sativa* subspecies *japonica* (Caicedo et al., 2007; Kumagai et al., 2010), are planted in large areas. Despite the continued debate regarding the origin(s) of domesticated *Oryza sativa* (Kovach et al., 2007; Sang and Ge, 2007; Molina et al., 2011; Huang et al., 2012; Civáñ et al., 2015; Huang and Han, 2015), both *indica* and *japonica* undoubtedly exhibit unique ecological distributions (Sang and Ge, 2007).

**Figure 1:**
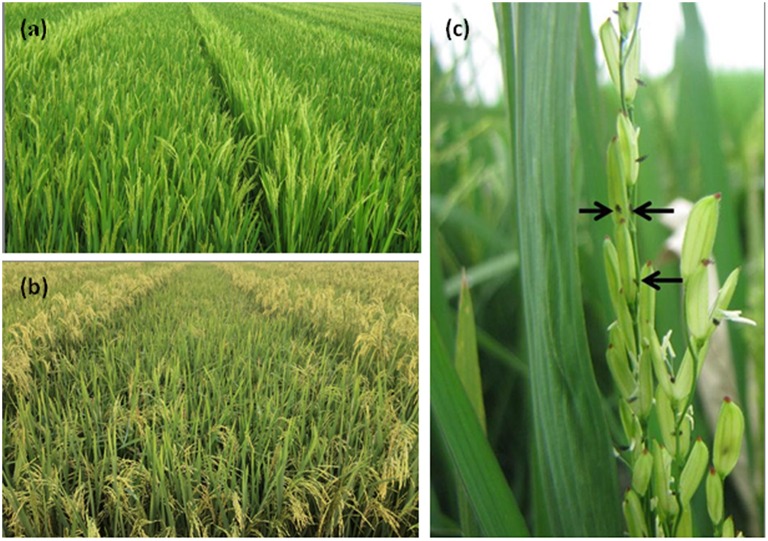
**Scene of F_**1**_ hybrid seed production in two different growth stages and exerted stigmas after the palea and lemma enclosed in a single plant in rice**. **(A)** Scene of F_1_ hybrid seed production in the heading stage. **(B)** Scene of F_1_ hybrid seed production in the filling stage. **(C)** Exerted stigmas (arrowheads) after the palea and lemma enclosed.

In China, rice is grown on 30 million hectares annually, and only India devotes a larger area to rice production. The annual rice production in China is 200.78 million tons, making China the largest rice producer in the world (Xie and Hardy, 2014). Each year, hybrid rice is planted on up to 50% of the total area in China devoted to rice production. The yield of hybrid rice cultivars is approximately 20% (1 ton per hectare) higher than that of conventional rice cultivars (Lu and Hong, 1999; Cheng et al., 2004). Despite the continuous improvements in cultivation techniques for F_1_ seed production over the last 10 years, the yield of hybrid rice seed production stagnated at 2.5 tons per hectare (Xie, 2009). A low stigma exsertion percentage is the main factor limiting further increases in the yield of F_1_ seed production in rice because it is a typical self-pollinating crop (Figure 1). To increase the percentage of rice stigma exsertion in rice, several studies have used QTL mapping to examine the genetic variation of stigma traits (Virmani and Athwal, 1973, 1974; Kato and Namai, 1987a; Virmani, 1994; Yamamoto et al., 2003; Uga et al., 2003a,b). To date, 50 QTLs that control stigma exsertion percentage have been mapped to all 12 rice chromosomes (Yamamoto et al., 2003; Uga et al., 2003a,b; Yu et al., 2006; Miyata et al., 2007; Hu et al., 2009; Yan et al., 2009; Li et al., 2014). Forty of the 50 QTLs were detected in the bi-parent derived segregating populations, and the remaining 10 were identified in the natural population. However, many environmental conditions, including high humidity, wind velocity, physical interruption, and low temperatures during the flowering period, influence stigma exsertion percentage (Beachell et al., 1938; Kato and Namai, 1987b), which may lower the accuracy of QTL mapping and complicate further studies. Therefore, to improve the outcrossing rate of the maternal parent, the trait stigma length (STL) can be more reliably measured than stigma exsertion in the mining of favorable alleles because STL is less influenced by external conditions than stigma exsertion percentage.

Several studies have shown that the stigma exsertion percentage is highly positively correlated with stigma length (Virmani and Athwal, 1973, 1974; Li and Chen, 1985; Kato and Namai, 1987a,b; Miyata et al., 2007). To the best of our knowledge, 23 QTLs that control stigma length have been detected, and these are distributed across all chromosomes with the exception of chromosomes 8 and 11 (Uga et al., 2003a, 2010; Yan et al., 2009; Liu et al., 2015). Most of these QTLs were identified using bi-parent-derived segregating populations. However, despite the availability of these scientific resources, linkage mapping is limited by the fact that only two alleles can be studied at any given locus in bi-parental crosses of inbred lines.

Recently, association mapping based on linkage disequilibrium (LD) has emerged as a popular method for mapping the loci responsible for natural variations, for mining natural genomic diversity, and for locating valuable genes (Zhu et al., 2008; Brachi et al., 2011; Weigel, 2012). In rice, association mapping has been used to exploit excellent alleles for many traits, including agronomic traits (Garris et al., 2005; Agrama et al., 2007; Huang et al., 2010, 2011; Zhao et al., 2011; Li et al., 2012; Vanniarajan et al., 2012; Dang et al., 2015; Yang et al., 2015), seed vigor traits (Cui et al., 2013; Dang et al., 2014; Rebolledo et al., 2015), and outcrossing traits (Yan et al., 2009). To the best of our knowledge, only one report (Yan et al., 2009) describes an association mapping-based study of stigma length.

The objectives of this study were to (1) evaluate the phenotypic diversity of stigma traits and the genetic architecture of the collection, (2) exploit elite alleles underlying stigma traits, and (3) predict the optimal cross combinations for improving stigma traits.

## Materials and methods

### Germplasm

A total of 227 *O*. *sativa* accessions were obtained from China (154), Vietnam (57), and Japan (16). The details of these accessions, including their origins and accession IDs, are summarized in Table S1. The seeds of all accessions were collected, stored and supplied by the State Key Laboratory of Crop Genetics, and Germplasm Enhancement at Nanjing Agricultural University.

### Field planting and trait measurement

Plants from the 227 accessions were grown in the paddy field of Jiangpu Experimental Station at the Nanjing Agricultural University in Nanjing (32°07′N, 118°64′E), Jiangsu Province, China, from May to October in 2013, and 2014. The field experiments conducted in each of the two consecutive years was treated as two independent environments. The field trials followed a completely randomized block design with two replicates per year. Each plot contained five rows, with eight plants in each row. The rows were spaced 20 cm apart, and each plant was spaced at a distance of 17 cm according to standard agronomic management practices.

**Figure 2:**
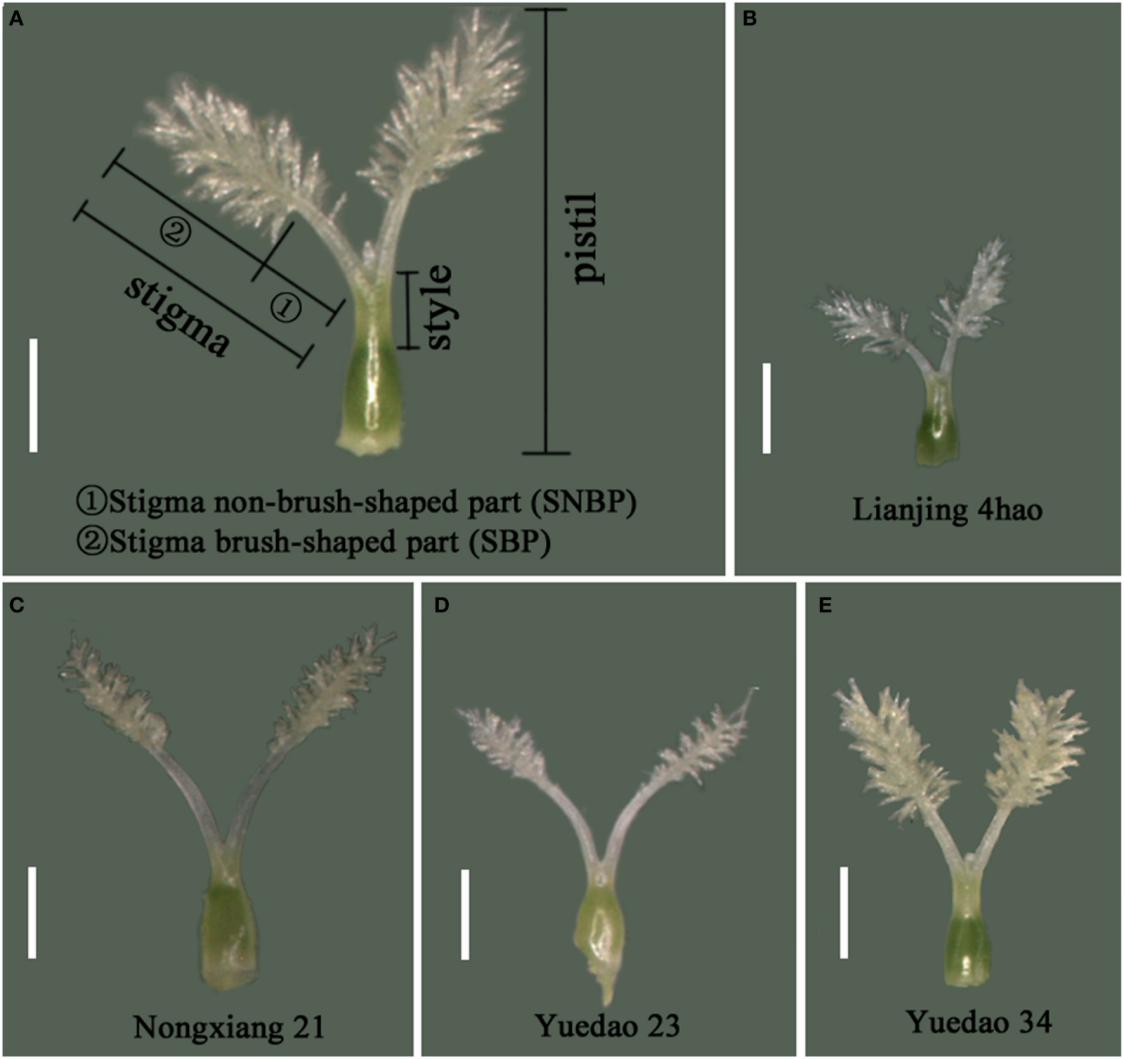
**Morphology of pistil and the stigma in rice**. **(A)** Names of rice pistil parts defined in this study. **(B)** Stigma morphology of Lianjing 4hao, with the minimum value of STL among 227 accessions. **(C)** Stigma morphology of Nongxiang 21, with the maximum value of STL among 227 accessions. **(D)** Stigma morphology of Yuedao 23. **(E)** Stigma morphology of Yuedao 34. The STL of Yuedao 23 and Yuedao 34 are equal. For Yuedao 23, SBPL is shorter than SNBPL. For Yuedao 34, SBPL is longer than SNBPL. Scale bar, 1 mm.

After pistil maturation, 10 spikelets were randomly collected from five plants of each accession prior to glume opening (approximately 10:00 a.m.). For each spikelet, the STL, SBPL, and SNBPL (Figure 2A) of the fertile flower were measured with a micrometer using a stereomicroscope (10 ×, MC50, Guangdong, China), and the average values served as the measurements for the accessions.

To understand the relationship between stigma traits and grain length (GL), fully filled grains were measured. In each year, five normally developed plants from each accession were harvested from the middle of each of the plots at the grain ripening stage and dried under natural conditions for GL investigation. Ten randomly selected grains (after the awns were removed) from each accession were lined up length-wise along an electronic digital Vernier caliper (http://www.guanglu.com.cn) to measure the grain length, and the average values served as the measurements for the accessions.

### SSR marker genotyping

Genomic DNA was extracted from young and healthy leaf blades of each accession approximately 3 months after germination using the methods described by Gross et al. (2009). According to the rice molecular map and microsatellite database published by Temnykh et al. (2000) and McCouch et al. (2002), 249 SSRs scattered on 12 chromosomes were selected. The SSR primers were synthesized by Shanghai Generay Biotech Co., Ltd. (Shanghai, China).

Each 10-μl PCR reaction contained 10 mM Tris-HCl (pH 9.0), 50 mM KCl, 0.1% Triton X-100, 1.5 mM MgCl_2_, 0.5 nM dNTPs, 0.14 pM forward primer, 0.14 pM reverse primer, 0.5 U of Taq polymerase, and 20 ng of genomic DNA. DNA amplification was performed using a PTC-100™ Peltier Thermal Cycler (MJ Research™ Incorporated, USA) under the following conditions: (1) denaturation at 94°C for 5 min; (2) 34 cycles of denaturation at 94°C for 0.5 min, annealing at 55–61°C for 1 min, and extension at 72°C for 1 min; and (3) a final extension at 72°C for 10 min. The PCR products were separated on an 8% polyacrylamide gel for 1 h at 150 V and visualized using silver staining. One pair of SSR markers was used to detect one locus, and each polymorphic band at the same marker locus in the population was recorded as one allele. After screening the PAGE products, the size of each band was determined using Quantity One software (Bio-Rad Company, USA).

### Population genetic structure

The genetic clusters in the 227 accessions were identified using STRUCTURE version 2.2 (Falush et al., 2007). This analysis was performed five times for each number of clusters (K) (from 2 to 10) with random starting points. We set the length of the burn-in period equal to 50,000 iterations and defined a run of 100,000 Markov Chain Monte Carlo (MCMC) replicates after burn in. A mean log-likelihood value over five runs at each K was used. If the mean log-likelihood value was positively correlated with the model parameter K, a suitable value for K could not be determined. In this situation, the optimal K value was determined through an *ad hoc* statistic, ΔK, based on the rate of change in [LnP(D)] between successive K values (Evanno et al., 2005). Non-admixed individuals in each genetic group were determined using a Q-matrix assignment greater than 0.9. A principal component analysis (PCA) was performed using the pcaMethods package (Stacklies et al., 2007) of R.2.11.1 (R Development Core Team, 2011) to examine the population structure. The first three principal components (PCs) were evaluated. The genetic distance was calculated based on 249 molecular markers using Nei's distance (Nei et al., 1983), and phylogenetic reconstruction was performed based on the neighbor-joining method implemented in PowerMarker version 3.25 (Liu and Muse, 2005). The tree used to visualize the phylogenetic distribution of the accessions and ancestry groups was constructed using MEGA version 4.0 (Tamura et al., 2007). The results from the three different approaches were then summarized and compared.

### Data analysis

All basic statistical analyses were performed using the SAS package (SAS Institute Inc., Cary, NC, USA). The broad-sense heritability (${\text{H}}_{\text{B}}^{2}$) was calculated based on the natural population through an analysis of variance using the formula ${\text{H}}_{\text{B}}^{2}=\sigma_{\text{g}}^{2}$/(σ${}_{\text{g}}^{2}$+σ${}_{\text{e}}^{2}$/n), where σ${}_{\text{g}}^{2}$ is the genetic variance, σ${}_{\text{e}}^{2}$ is the error variance, and n is the number of replicates.

The polymorphic information content (PIC) was used to measure the probability that two randomly selected alleles from a population were distinct. The number of alleles per locus, the gene diversity, and the PIC values were determined using PowerMarker version 3.25 (Liu and Muse, 2005).

The average standardized individual allele size of the SSRs was determined by calculating the mean standardized size of 249 SSR loci following the method reported by Vigouroux et al. (2003). This standardization ensured that each locus contributed equally to the average individual size of the genome.

### Linkage disequilibrium

The D′ value (Farnir et al., 2000) was calculated with TASSEL 3.0 software using 1000 permutations and was used to measure the level of linkage disequilibrium (LD) between loci (Bradbury et al., 2007). Before the association analysis, rare alleles with allele frequencies of 5% or less were removed from the dataset. According to the level of LD and the genetic distance among markers with intrachromosomal combinations, the regression equation of the LD as a function of changes in the genetic distance was calculated through a regression analysis. A LD decay plot was drawn to observe the relationship between LD and genetic distance.

### Association mapping

A Mixed Linear Model (MLM) analysis, which can significantly reduce spurious marker-trait associations (type I errors showing false positives) resulting from the population structure, was performed using TASSEL 3.0 to calculate the associations between traits and markers (Bradbury et al., 2007). The matrices Q and K were used as covariants in the MLM analysis. The Q matrix was adapted from the analysis results obtained from Structure 2.2. The K matrix (kinship matrix) was obtained from the results of the relatedness analysis using SPAGeDi (Hardy and Vekemans, 2002). According to the correction method published by Benjamini and Hochberg (1995), a false discovery rate (FDR) of 0.05 was used as a threshold for significant associations. Based on the identified association locus, the “null allele” (non-amplified allele) was used to determine the phenotypic effects of other alleles (Breseghello and Sorrells, 2006).

### Phylogeography

To detect a phylogeographic signal in *O. sativa*, we divided the distribution range into six regions: Vietnam (8°30′–23°22′N, 102°8′–109°24′E), Southern China (20°45′–30°08′N, 97°31′–124°34′E), Eastern China (27°12′–35°20′N, 116°18′–123°00′E), Northern China (31°23′–42°37′N, 110°21′–119°45′E), Northeastern China (38°43′–53°33′N, 118°53′–135°05′E) and Japan (30°38′–44°09′N, 130°49′–145°15′E). This delimitation was based on the latitude of each region. We performed a locus-by-locus analysis of molecular variance (AMOVA) (Weir and Cockerham, 1984) based on genetic groups delimited by the Bayesian clustering method in the program Arlequin 3.5 (Excoffier and Lischer, 2010) to statistically verify the geographical structure using SSR and standard multi-locus frequency data.

## Results

### Phenotypic variations in the rice germplasm and correlations among traits

The mean value, standard deviation, skewness, and kurtosis for stigma traits measured in 227 rice varieties were calculated (Table 1). A significantly positive correlation was found between stigma traits in 2013 and 2014 (Figure S1). The materials exhibiting the minimum and maximum values are shown in Figures 2B,C. The stigmas exhibited longer SNBPLs and shorter SBPLs in some accessions (Figure 2D), and these trends were reversed in other accessions (Figure 2E). The average STL, SBPL, and SNBPL values of 227 accessions over the 2 years were 1.8 mm, 1.1 mm, and 0.7 mm, respectively. The phenotypic data for the STL, the SBPL, and the SNBPL in the studied population followed normal distributions (Table 1). Table 1 also shows that the average SBPL was greater than the average SNBPL. The broad-sense heritability values for the STL, SBPL, and SNBPL traits averaged over 2 years were 93.8, 92.8, and 88.6%, respectively. Furthermore, the three stigma traits exhibited significant positive correlations. The correlation coefficients between the STL and SBPL, the STL and SNBPL, and the SBPL and SNBPL were 0.803, 0.787, and 0.264, respectively (Table 2).

**Table 1:** **Phenotypic characteristics for stigma length and grain length traits in 227 rice accessions across 2 years**.

**Trait**	**Year**	**Maximum**	**Minimum**	**Mean**	**Standard deviation^a^**	**Skewness**	**Kurtosis**	**Heritability in the broad sense %**
Stigma length (mm)	2013	2.89	1.28	1.79	0.29	0.74	0.25	92.6
	2014	2.97	1.32	1.82	0.27	0.62	0.65	94.9
Stigma brush-shaped part length (mm)	2013	1.65	0.58	1.08	0.18	0.34	0.25	94.4
	2014	1.58	0.62	1.07	0.17	0.14	0.05	91.2
Stigma non-brush-shaped part length (mm)	2013	1.76	0.17	0.71	0.20	0.36	2.55	85.6
	2014	1.46	0.21	0.76	0.16	0.27	1.56	91.5
Grain length (mm)	2013	12.91	6.59	8.24	1.16	1.65	2.88	98.7
	2014	12.73	6.45	8.16	1.12	1.70	3.56	97.9

a *The size of sample is 227 accessions*.

**Table 2:** **General linear correlation analysis for stigma length and grain length traits**.

	**STL**	**SBPL**	**SNBPL**	**GL**
STL	-			
SBPL	0.80^**^	-		
SNBPL	0.79^**^	0.26^**^	-	
GL	0.71^**^	0.48^**^	0.65^**^	-

** *Significant at P < 0.01*.

The highest average GL value over 2 years was 12.8 mm, the lowest average GL value over 2 years was 6.5 mm, and a skewed distribution was observed based on the skewness and kurtosis statistics (Table 1). The broad-sense heritability value averaged over 2 years was 98.3%. The correlation coefficients between the GL and STL, the GL and SBPL, and the GL and SNBPL were 0.710, 0.477, and 0.654, respectively (Table 2).

### Genetic variation in the rice germplasm

A marker analysis of the 227 accessions using 249 SSR molecular markers resulted in the detection of 2072 alleles. The numbers of alleles ranged from two (at locus RM7163_Chr11) to 18 (RM162_Chr6), with an average of 8.32 alleles per locus (Table S2). The genetic diversity averaged 0.699 and ranged from 0.083 (RM7163_Chr11) to 0.894 (RM162_Chr6) (Table S2). The PIC had a mean value of 0.668, ranged from 0.050 (RM7163_Chr11) to 0.885 (RM162_Chr6), and was mainly distributed between 0.509 and 0.809 (Table S2). A total of 210 markers (84%) were highly informative (PIC > 0.5), 27 (11%) were moderately informative (0.5 > PIC > 0.25), and 12 (5%) were slightly informative (PIC < 0.25).

### Population structure

The highest delta K values from the STRUCTURE analysis were obtained for eight clusters (Figure S2). The population structure data based on the Q matrix for each accession are summarized in Table S1, and the 227 accessions could be divided into eight subpopulations, viz. Group 1 to Group 8 and an admixed group (Figure S3A). Group 1 contained 38 accessions, which were modern improved varieties obtained mainly from Eastern China. Group 2 contained 45 accessions, which were mainly from Vietnam. Group 3 contained 20 accessions, and these were mainly obtained from Japan. Group 4 included 32 accessions, which consisted of landraces from Eastern China. Group 5 contained 21 accessions, which were mainly from Northern China. Group 6 contained 25 accessions, and these were mainly obtained from Southern China. Group 7 included 30 accessions, which were mainly from Northeastern China. Group 8 only contained four accessions obtained from North and Northeastern China. The admixed group contained the remaining 12 accessions.

Although the non-admixed individual cluster contained some admixed individuals, the neighbor-joining tree indicated that each of the eight groups corresponded to a distinct branch on the tree (Figures S3B, S4). These admixed individuals tended to be genetically similar to the group corresponding to their branch but exhibited a small amount of introgression from other groups.

The PCA results were essentially consistent with the STRUCTURE findings. The eight genetic groups were distinct in the principal component space of the three major PCs, whereas some individuals appeared mixed (Figure S3C). The three major PCs identified from the PCA explained 31.8% (11.4, 10.3, and 10.1%) of the total variance.

The following analysis did not include the accessions in the admixed group or Group 8 because Group 8 contained only four accessions.

### Geography-based directional evolution in the allele length of subpopulations

**Figure 3:**
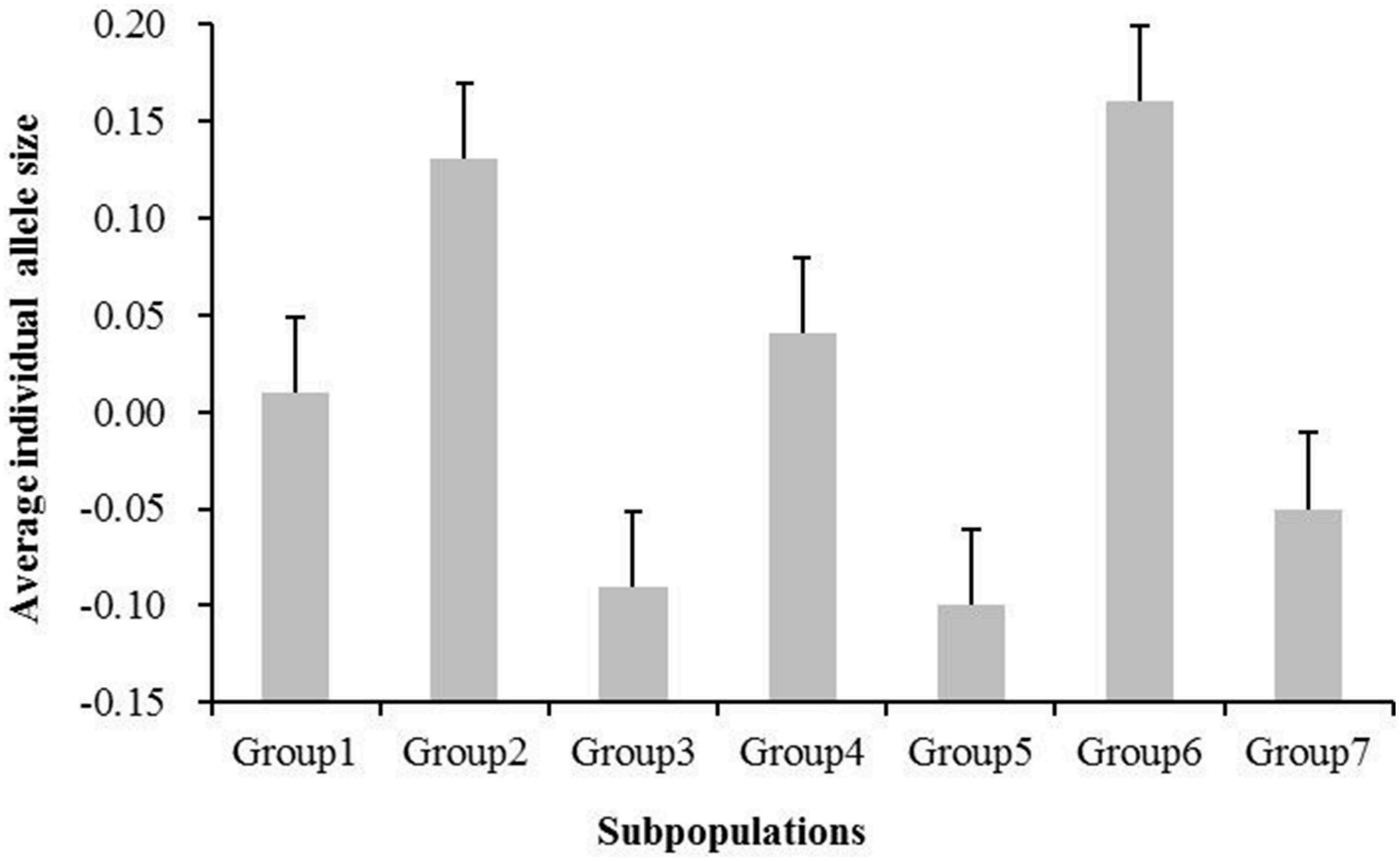
**Average individual microsatellite allele size for the seven subgroups (Groups 1–7)**. The standard error is presented.

To test whether the microsatellite size exhibited directional evolution in rice, we compared the average allele sizes in the geographically derived groups (Groups 1–7). Figure 3 shows the average individual microsatellite allele size for these seven groups. No significant differences were detected between Groups 1 and 4 (*P* > 0.05, two-tailed *t*-test), Groups 2 and 6 (*P* > 0.05, two-tailed *t*-test), or Groups 3 and 5 (*P* > 0.05, two-tailed *t*-test), whereas the rest groups exhibited significant differences, with *P*-values less than 0.001. The average individual SSR allele size in the subgroups from low latitudes (Groups 2 and 6) was 0.14, which is greater than the value obtained for subgroups from high latitudes (-0.04; Figure 3). These data indicate a decrease in allele size from south to north in the geographically derived groups.

### Linkage disequilibrium

A total of 18,120 of 30,876 pairs, including both interchromosomal, and intrachromosomal combinations, showed significant LD (based on D′, *P* < 0.05). Of these 18,120 pairs, 1530 were intrachromosomal pairs of SSR loci, which corresponds to a rate of 8.4%. The extent of LD was assessed for each of the seven groups (Table 3). For the average D′, Group 1 showed the lowest LD (0.399), whereas Group 5 exhibited the highest LD (0.468).

**Table 3:** **Comparison of D′ values for pair-wise SSR loci in seven subpopulations**.

**Cluster**	**No. of LD^a^ locus pairs**	**Ratio^b^ (%)**	**Frequency of D′^c^ value (*P* < 0.05)**					**Mean of D′**
			**0–0.2**	**0.2–0.4**	**0.4–0.6**	**0.6–0.8**	**0.8–1.0**	
Group 1	1713	9.05	41	45	39	16	14	0.399
Group 2	1455	21.24	82	68	68	26	65	0.459
Group 3	1611	6.70	33	36	15	9	15	0.407
Group 4	772	13.60	28	26	22	23	6	0.412
Group 5	658	13.53	15	26	20	18	10	0.468
Group 6	1108	10.92	22	42	16	21	20	0.464
Group 7	425	20.24	21	26	14	8	17	0.449

a *LD means linkage disequilibrium*.

b *Ratio between the number of intrachromosomal significant LD locus pairs and total number of significant LD locus pairs*.

c *D′ means standardized disequilibrium coefficients (Farnir et al., 2000)*.

**Figure 4:**
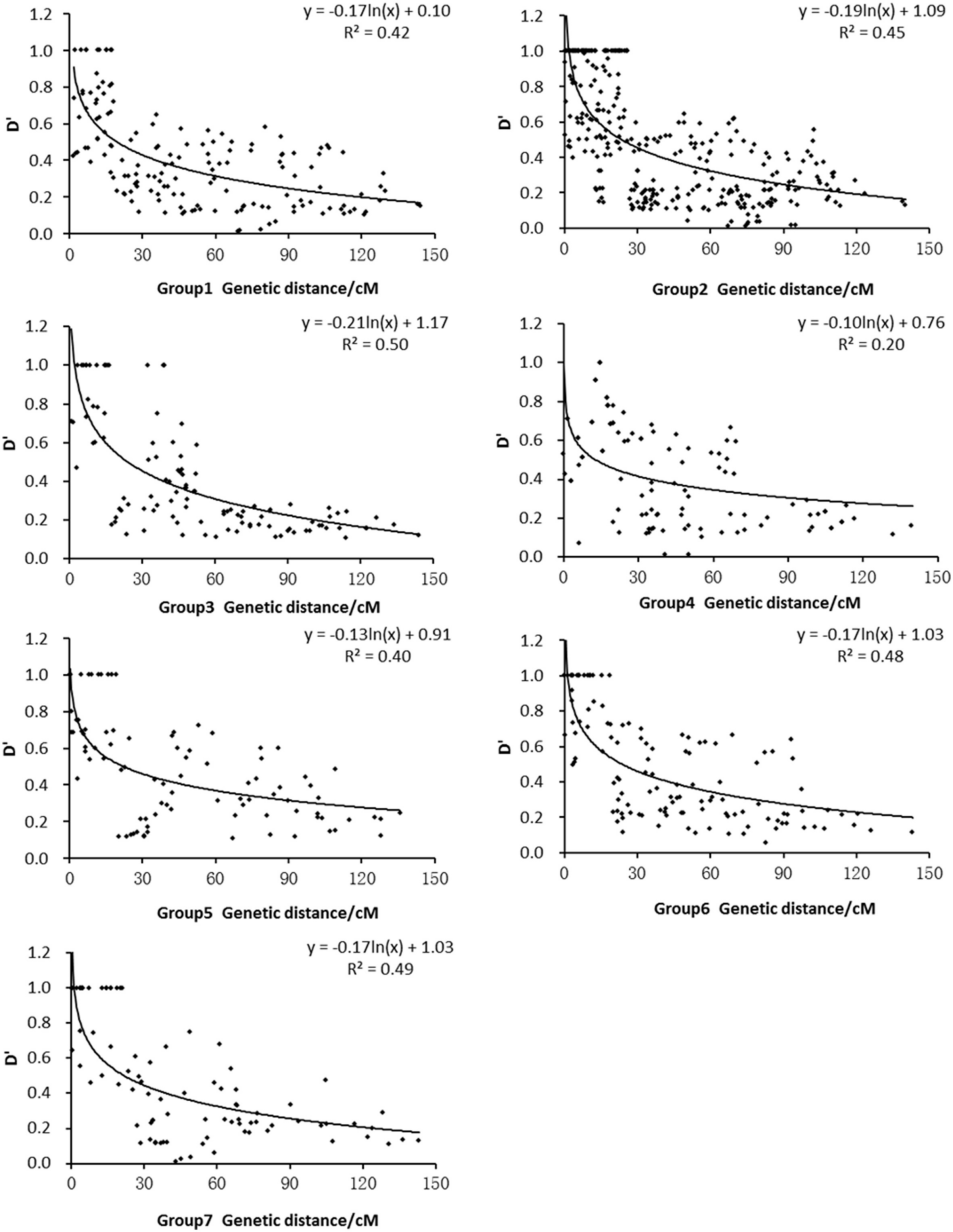
**Relationship between D′ value and genetic distance of syntenic marker pairs in subpopulations**.

We used the D′ value corresponding to the intrachromosomal SSR loci and the genetic distance in each subpopulation to draw the attenuation map. Figure 4 shows that the D′ values decayed as the genetic distance (cM) increased. A regression analysis between the D′ value and the genetic distance of syntenic marker pairs revealed that the seven subpopulation genomes fitted the equation *y* = *b* ln*x* + *c*. The minimum distances of LD decay for Groups 1, 2, 3, 4, 5, 6, and 7 were 19.5 cM, 23.2 cM, 24.1 cM, 12.9 cM, 22.2 cM, 22.8 cM, and 21.7 cM, respectively. Thus, among the seven subpopulations, Group 4 exhibited the highest decay velocity with the shortest decay distance, whereas Group 3 demonstrated the lowest decay velocity.

### Genetic differentiation across subpopulations

The average *F*_ST_ among the seven subpopulations was 0.672, with the *F*_ST_ for each locus ranging from 0.197 for RM333_Chr 10 to 0.984 for RM6361_Chr 2. A results of a pairwise comparison based on the values of *F*_ST_ can be interpreted as the standardized population distances between two subpopulations. The pairwise *F*_ST_ values obtained in the present study ranged from 0.162 (between Groups 2 and 6) to 0.832 (between Groups 2 and 7), with an average value of 0.679 (Table 4). The AMOVA results indicated that 67.88% of the total genetic variation occurred between the subpopulations, whereas 32.12% occurred within the subpopulations (Table S3). These results indicate the existence of a high degree of genetic differentiation across the seven subpopulations.

**Table 4:** **Pairwise estimates of ***F***_**ST**_ based on 249 SSR loci among the seven model-based subpopulations**.

**Cluster**	**Group 1**	**Group 2**	**Group 3**	**Group 4**	**Group 5**	**Group 6**	**Group 7**
Group 1	-						
Group 2	0.640	-					
Group 3	0.715	0.604	-				
Group 4	0.723	0.641	0.752	-			
Group 5	0.719	0.633	0.750	0.748	-		
Group 6	0.615	0.162	0.677	0.727	0.731	-	
Group 7	0.625	0.832	0.779	0.810	0.686	0.684	-

### Significant marker-trait association loci detected across the entire population

A marker-trait association analysis based on an MLM revealed that six markers located on chromosomes 1, 2, 4, and 6 were associated with the STL (Table 5). The phenotypic variation explained (PVE) ranged from 2.9 to 12.5%. RM5753_Chr 6, which resides at 124.4 cM, had the highest PVE values for the STL, namely 12.5% in 2013, and 8.9% in 2014 (Table 5). Two markers distributed on chromosomes 4 and 6 were associated with the SBPL (Table 5), and of these, RM136_Chr 6 had the highest average PVE of 6.5% over the 2 years. Seven markers distributed on chromosomes 1, 2, 4, 6, and 11 were associated with the SNBPL (Table 5), and their PVE values ranged from 3.1 to 12.9%.

**Table 5:** **Marker-trait associations with ***P***-value less than 0.05, their equivalent false discovery rate probability (FDR), proportion of phenotypic variance explained (PVE), marker position on chromosome derived from 249 markers and 211 rice accessions**.

**Trait**	**Markers**	**Chr**.	**Position/cM**	**2013**			**2014**		
				***P*-value**	**PVE**	**FDR**	***P*-value**	**PVE**	**FDR**
STL	RM5389	1	142.4	9.00E-04	0.107	6.67E-03	9.60E-03	0.076	1.11E-02
	RM450	2	122.8	4.31E-02	0.049	4.67E-02	3.27E-02	0.054	3.33E-02
	RM7598	2	126.4	1.74E-04	0.100	3.33E-03	1.55E-03	0.075	5.56E-03
	RM280	4	128.9	5.24E-03	0.038	1.33E-02	1.45E-02	0.029	1.67E-02
	RM7579	6	84.5	1.52E-02	0.051	3.33E-02	4.44E-02	0.038	4.91E-02
	**RM5753**^a^	6	124.4	4.86E-03	0.125	1.00E-02	2.37E-02	0.089	2.78E-02
SBPL	RM280	4	128.9	3.02E-02	0.022	1.67E-02	2.50E-02	0.032	2.50E-02
	**RM136**	6	53.0	3.69E-02	0.068	4.17E-02	3.74E-02	0.061	4.17E-02
SNBPL	RM5389	1	142.4	3.31E-03	0.088	7.14E-03	2.06E-02	0.067	2.08E-02
	RM450	2	122.8	8.55E-03	0.070	2.14E-02	2.23E-03	0.084	8.33E-03
	RM7598	2	126.4	8.55E-04	0.086	3.57E-03	1.22E-03	0.082	4.17E-03
	RM280	4	128.9	7.18E-03	0.035	1.79E-02	1.10E-02	0.032	1.25E-02
	**RM559**	4	129.6	4.31E-02	0.032	4.64E-02	4.55E-02	0.031	4.58E-02
	**RM5753**	6	124.4	6.41E-03	0.112	1.43E-02	3.48E-02	0.082	3.75E-02
	**RM6327**	11	1.7	3.65E-03	0.129	1.07E-02	4.07E-02	0.081	4.17E-02
GL	**RM128**	1	126.5	1.39E-03	0.114	1.47E-02	3.05E-04	0.141	1.25E-02
	**RM5389**	1	142.4	3.04E-04	0.124	1.18E-02	6.11E-04	0.116	1.56E-02
	RM450	2	122.8	6.81E-06	0.156	5.88E-03	8.77E-07	0.182	6.25E-03
	RM7598	2	126.4	1.05E-08	0.207	2.94E-03	1.99E-09	0.229	3.13E-03
	RM282	3	55.8	1.26E-02	0.090	2.65E-02	4.08E-03	0.111	2.19E-02
	RM6712	3	158.2	4.35E-02	0.055	4.41E-02	2.52E-02	0.064	4.06E-02
	RM6314	4	41.5	3.52E-02	0.058	4.12E-02	2.87E-02	0.056	4.38E-02
	**RM280**	4	128.9	2.79E-02	0.024	3.82E-02	3.44E-02	0.022	4.69E-02
	RM136	6	53.0	2.38E-02	0.081	3.24E-02	1.03E-02	0.094	2.81E-02
	RM2530	7	53.4	1.87E-03	0.106	1.76E-02	1.04E-03	0.116	1.88E-02
	RM6976	8	92.2	1.07E-02	0.087	2.35E-02	1.96E-02	0.077	3.44E-02
	RM1125	10	46.8	1.04E-02	0.106	2.06E-02	6.29E-03	0.112	2.50E-02
	**RM6327**	11	1.7	6.65E-05	0.218	8.82E-03	2.18E-05	0.229	9.38E-03

a *Markers in bold face means the new loci identified in this study*.

Thirteen markers distributed on chromosomes 1, 2, 3, 4, 6, 7, 8, 10, and 11 were associated with the GL (Table 5), and their PVE values ranged from 2.2 to 22.9%. RM6327_Chr 11, which resides at 1.7 cM, had the maximum PVE for the GL, specifically 21.8% in 2013, and 22.9% in 2014 (Table 5).

Using the entire set of accessions, we identified 28 markers associated with stigma traits and grain length, including six markers associated with the STL, two related to the SBPL, seven associated with the SNBPL, and 13 associated with the GL. Nineteen of the 28 associations were in regions where the QTL associated with the given trait had been identified by Redoña and Mackill (1998), Tan et al. (2000), Uga et al. (2003a), Aluko et al. (2004), Li et al. (2004), Agrama et al. (2007), Bai et al. (2010), Uga et al. (2010), Wang et al. (2011, 2012), Dang et al. (2015) (http://www.gramene.org/); these QTLs are listed in Table S4. Nine loci identified in this study, including one for the STL, one for the SBPL, three for the SNBPL, and four for the GL, are novel (Table 5).

The marker RM280 was co-associated with the STL, SBPL and SNBPL. Five markers, i.e., RM5389, RM450, RM7598, RM280, and RM5753, were co-associated with the STL and SNBPL. Four markers, namely RM5389, RM450, RM7598, and RM280, were co-associated with the GL and STL and with the GL and SNBPL. Two markers, RM280 and RM136, were co-associated with the GL and SBPL (Table 5).

### Discovery of elite alleles

The alleles with positive effects identified in this study were considered elite alleles for all four traits measured. A summary of the elite alleles and their typical carrier materials is shown in Table S5. The total numbers of elite alleles detected across the entire population for the STL, SBPL, and SNBPL were 11, 5, and 12, respectively. The allele RM450-135 bp showed the greatest phenotypic effect (0.235 mm) on the STL, and its typical carrier accession was Yuedao 32. The allele RM136-200 bp showed the greatest phenotypic effect (0.111 mm) on the SBPL, and its typical carrier accession was Yuexiangzhan. The allele RM450-135 bp showed its greatest phenotypic effect (0.233 mm) on the SNBPL, and its typical carrier accession was Yuedao 32.

A total of 30 elite alleles were detected for the GL. The allele RM136-200 bp exerted the greatest phenotypic effect (1.612 mm) on the GL, and its typical carrier accession was Yuexiangzhan (Table S5).

Correlations between the measured traits were observed, and the STL was significantly positively correlated with the SBPL and SNBPL. Furthermore, the GL was also significantly positively correlated with the STL, SBPL, and SNBPL. We identified one SSR marker co-associated with the STL and the SBPL, and the allele RM280-175 bp in this SSR marker locus simultaneously increased the phenotypic effect values of the STL and SBPL (Table 5 and Table S5). We identified five SSR markers co-associated with the STL and SNBPL, and the alleles RM5389-120, RM450-135, RM450-155, RM7598-115, RM280-175, RM5753-200, and RM5753-205 bp at these marker loci simultaneously increased the phenotypic effect values of the STL and SNBP (Table 5 and Table S5). We also identified four SSR markers co-associated with the STL and GL, and the alleles RM5389-120, RM450-135, RM450-155, RM7598-115, and RM280-175 bp at these marker loci simultaneously increased the phenotypic effect values of the STL and GL (Table 5 and Table S5). We further identified one SSR marker co-associated with the STL, SBPL, SNBPL, and GL, and the allele RM280-175 bp at this marker locus simultaneously increased the phenotypic effect values of the STL, SBPL, SNBPL, and GL (Table 5 and Table S5). These co-associated alleles have the correct sign with respect to trait correlations, and these data illustrate the genetic basis of trait correlations.

### Optimal cross designs for improving target traits

Based on the number of elite alleles that could be substituted into an individual plant and the expected phenotypic effects of the elite alleles that could be pyramided, the top five cross combinations for improving the STL, SBPL, SNBPL, and GL are proposed (Table S6). The elite alleles carried by the parents in excellent crosses and the corresponding phenotypic effects are listed in Table S7. Figure S5 shows the excellent parents in the superior cross for stigma traits. Certain accessions were found repeatedly in these proposed parental combinations (e.g., Yuedao 32 emerged four times in the combinations for the STL), indicating that these accessions possess unique elite alleles.

## Discussion

All three analyses, STRUCTURE, PCA and the neighbor-joining method, uncovered the same pattern of eight very distinct genetic groups in the samples of *O. sativa* investigated (Figure S3). Most accessions of these groups clearly belonged in unique subgroups, and minor accessions belonged in admixed subgroup (Table S1). In addition, the population structure was observed to be tied to the geographical origin, e.g., the accessions from Vietnam essentially clustered into Group 2, and the accessions from Northeastern China primarily clustered into Group 7. The distinctive geographical origins corresponding to the differences in ecological environments may have been partially responsible for the genetic differentiation. These findings demonstrate that natural populations of *O*. *sativa*, which is a self-pollinating species, show clear population subdivisions and a high amount of genetic diversity based on SSR markers resulting from historical mixtures among the subgroups.

No significant differences were found between Group 1 and Group 4, between Group 2 and Group 6, or among Group 3, Group 5, and Group 7 in the average standardized individual SSR allele sizes (Figure 3). The average standardized individual SSR allele sizes in Group 1 and Group 4 (mainly from Eastern China) is smaller than that in Group 2 and Group 6 (mainly from Southern China), but larger than that in Group 3, Group 5, and Group 7 (mainly from Northern China). This result indicates a tendency for directional evolution in SSRs during improvement of *Oryza sativa* from low (warmer climate) to high latitudes (cooler climate).

The average number of alleles per locus was 8.32 among the 227 accessions, which were genotyped using 249 markers (Table S2). This allele number per locus was higher than the values reported by Cho et al. (2000), Jain et al. (2004), Garris et al. (2005), Agrama et al. (2007), Jin et al. (2010), and Vanniarajan et al. (2012) but lower than those reported by Thomson et al. (2007), Borba et al. (2009), Li et al. (2012), and Dang et al. (2014, 2015). The average polymorphic information content (PIC) value obtained in this study was 0.668, which is the same as that reported by Thomson et al. (2007) and higher than those obtained in all previously published studies of rice populations (Ordonez et al., 2010; Vanniarajan et al., 2012) with the exception of those reported by Li et al. (2012), Dang et al. (2014, 2015) and Borba et al. (2009) (0.71, 0.71, and 0.75, respectively). The wide range of genetic diversity observed in this study among the accessions with a broad geographical origin makes the set of accessions included in this study one of the best collections for mining valuable genes in rice.

The studies conducted by Olsen et al. (2006), Mather et al. (2007) and Rakshit et al. (2007) using DNA sequences indicated that the LD decays at 1 cM or less in rice. In contrast, other studies using SSR markers, such as those performed by Agrama et al. (2007), Vanniarajan et al. (2012) and Dang et al. (2014, 2015), indicated that LD decays at 20–30 cM, 20–30 cM, 10–80 cM, and 10–30 cM, respectively. The analysis of the LD levels of Groups 1 to 7 in this study revealed values that were similar to those reported by Vanniarajan et al. (2012) and Dang et al. (2015). These results suggest that the extent of LD varies by genomic region (Mather et al., 2007), rice accession (Agrama and Eizenga, 2008), and marker. Moreover, Group 4 exhibited the fastest decay velocity, followed by Group 1, and Groups 2, 3, 5, 6, and 7 exhibited the lowest decay velocities in this study (Figure 4). The fast LD decay of Group 4 is mainly attributable to the high outcrossing rate of landraces. The global high LD in cultivated rice is mainly attributable to selfing and strong bottleneck (Mather et al., 2007; Zhu et al., 2007).

The *F*_ST_ values of the non-admixed individuals from the seven subgroups are exceedingly high (0.672 by AMOVA) compared with those observed in large-scale samples of other species, such as *Oryza rufipogon* (0.1, Huang et al., 2012), *Arabidopsis thaliana* (0.2, Long et al., 2013) and *Setaria viridis* (0.49, Huang et al., 2014), indicating substantial differentiation among subgroups, and genomic differentiation among species. The high value of *F*_ST_ for the seven subgroups in our study identified large differences between the accessions (Table 4). The markers with higher *F*_ST_ values had the greatest resolution power and produced more consistent genetic distance estimates, which is consistent with the results obtained from an analysis of rice conducted by Agrama et al. (2007) and a study of a human population performed by Watkins et al. (2003). The significant *F*_ST_ values suggest a real difference among the subgroups, and these differences might be used to predict heterotic crosses for improving yield in hybrid breeding.

We also found that the marker RM7598_Chr2, which co-associated with the STL and SNBPL, is located in the region (20,044,821—35,072,135 bp) in which a QTL for stigma exsertion was identified by Hu et al. (2009). The marker RM280_Chr4, which co-associated with the STL, SBPL, and SNBPL, is in the region (17,689,612—26,137,600 bp) in which a QTL for stigma exsertion was identified by Yamamoto et al. (2003). These results confirm the close relationship between stigma traits (STL, SBPL, and SNBPL) and stigma exsertion.

In addition, certain loci were mapped close to gene resolution, e.g., RM282 was close to GL3 (Wang et al., 2012), indicating that association analyses of rice accessions can provide an effective approach for gene identification.

Using the entire population to mine elite alleles can maintain information integrity (Dang et al., 2015). In this study, 11 elite alleles for the STL were mined at the six identified loci. Among these alleles, 18.2% were carried by accessions collected from Northeastern China, 27.3% were carried by accessions from Central China, and 54.5% were carried by accessions from Vietnam. Similarly, certain unique elite alleles for the SBPL, SNBPL, and GL were identified in various accessions. These results suggested that during the process of rice evolution from southern to northern latitudes, the loss of certain SSR alleles is likely caused by domestication bottleneck, whereas others were retained or appear for the first time in modern cultivars. For example, RM7598-115 bp is common among Vietnam accessions but is not found in Northeastern China accessions, whereas the allele RM7598-95 bp is found only in Northeastern China accessions.

For the STL trait, the broad-sense heritability averaged over the 2 years was 93%. Among the six SSR-associated markers detected for the STL, RM5753_Chr 6 exhibited the largest PVE (12.5% in 2013 and 8.9% in 2014). Of the two elite alleles found at this marker locus, RM5753-200 bp presented the largest phenotypic effect value (0.162 mm). This elite allele was carried by 57 accessions, and Yuedao 32 was the typical carrier material. Thus, the crosses described in Table S6 could significantly improve the STL.

For the SBPL trait, the broad-sense heritability averaged over the 2 years was 93%. Of the two SSR-associated markers detected for the SBPL, RM136_Chr 6 had the largest PVE (6.8% in 2013 and 6.1% in 2014). Among the four elite alleles found at this marker locus, RM136-200 bp presented the largest phenotypic effect value (0.111 mm). This elite allele was carried by 16 accessions, and Yuexiangzhan was the typical carrier material. Thus, the crosses described in Table S6 could significantly improve the SBPL.

The broad-sense heritability for the SNBPL trait averaged over the 2 years was 89%, which is also high. Among the seven SSR markers associated with the SNBPL, RM6327_Chr 11 had the largest PVE (12.9% in 2013 and 8.1% in 2014), and two elite alleles—RM6327-180 bp and RM6327-200 bp—were found at this marker locus. Thus, the SNBPL might be improved by the crosses listed in Table S6.

For the GL, the broad-sense heritability averaged over the 2 years was 98%, which is a considerably high value. Thus, the expected improvements in the GL could be obtained by marker-assisted selection. Among the 13 SSR-associated markers detected for the GL, RM6327_Chr 11 presented the largest PVE (21.8% in 2013 and 22.9% in 2014). Of the two elite alleles found at this marker locus, RM6327-190 bp exhibited the largest phenotypic effect value (0.787 mm). This elite allele was carried by 19 accessions, and Yuexiangzhan was the typical carrier material. Thus, the crosses described in Table S6 could significantly improve the GL. Because the STL was positively correlated with the GL, increases in the GL will also improve the STL. In addition, the STL could also be improved independently from the GL because the determinant coefficient between the STL and the GL was approximately 0.5.

If the target trait must be improved further, the best elite alleles could be pyramided into one cultivar through multi-round crossing. For example, 11 elite alleles were detected for STL, and the six best elite alleles could be pyramided or substituted by the combination of the accessions Yuzhenxiang, Yuedao 32, Yuedao 100, Yuexiangzhan, and Nongxiang 18 (Table S6).

## Author contributions

DH planned and designed the research; XD, QL, CB, and YL. Performed the field experiment; XD, EL, QL, and CB. Conducted the molecular experiment; XD and EL. Analyzed the data; XD. Wrote the manuscript; and DH. Revised the manuscript. All authors read and approved the manuscript.

### Conflict of interest statement

The authors declare that the research was conducted in the absence of any commercial or financial relationships that could be construed as a potential conflict of interest.

## Supplementary material

Figure S1 **Correlation of stigma traits between 2013 and 2014**. A total of 227 rice accessions were grown in the same field in 2013 and 2014 growing season. **(A)** Correlation of STL between 2013 and 2014. **(B)** Correlation of SBPL between 2013 and 2014. **(C)** Correlation of SNBPL between 2013 and 2014. ^**^Significantly correlation at the *P* < 0.01 (two-tailed).

Figure S2 **Changes of the mean LnP (K) (A) and **Δ**K (B). (A)** A graph with mean LnP (K) on *Y*-axis and number of subgroups on *X*-axis; **(B)** A graph with ΔK on *Y*-axis and number of subgroups on *X*-axis.

Figure S3 **Population structure of 227 ***Oryza Sativa***. (A)** STRUCTURE result. The panel is a result of *K* = 8 (highest likelihood result among replicates). Each accession is represented by a vertical bar. The colored subsections within each vertical bar indicate membership coefficient (Q) of the accession to different clusters. Identified subpopulations are Group1 (red color), Group2 (green color), Group3 (navy blue color), Group4 (yellow color), Group5 (purple color), Group6 (light blue color), Group7 (brown color) and Group8 (caramel color). Accessions marked by group name are considered non-admixed (more than 0.9 assignment to one group in STRUCTURE analysis with *K* = 8). **(B)** Result of principal component analysis. Each point corresponds to an individual, and the different color slices correspond to the group assignment matrix (Q matrix) of the STRUCTURE result for *K* = 8. Numbers in parentheses beside each axis denote the amount of variance explained by that axis. **(C)** Neighbor-joining tree. The different colored groups approximately correspond to Groups 1–8. Colored branches represent the non-admixed individuals within each corresponding group. Gray branches respent admixed individuals (< 0.9 assignment assigned to any group in STRUCTURE analysis with *K* = 8).

Figure S4 **Neighbor-joining tree constructed from Nei's (1983) genetic distance of 249 SSRs**. Each branch is corresponding to each accession ID.

Figure S5 **Rice stigma morphology of the 6 elite parents included in the elite crosses predicted**.

Supp. Table S1 **Accessions used and corresponding values of the Q matrix in this study**.

Supp. Table S2 **Summary statistics for the 249 SSR markers used in this study**.

Supp. Table S3 **Locus-by-locus AMOVA for ***O. sativa*** based on Structure groups**.

Supp. Table S4 **Positive elite alleles, phenotypic effect value and typical materials for stigma traits and grain length**.

Supp. Table S5 **Parental combinations and numbers of elite alleles after combinations predicted from association mapping of stigma traits and grain length**.

Supp. Table S6 **Elite alleles carried by the superior parents for stigma traits and grain length and corresponding phenotypic effect**.

Supp. Table S7 **The list for QTLs identified from this study and shared in previous studies**.
