# Supplementary figures and images for: QTL Detection and Elite Alleles Mining for Stigma Traits in Oryza sativa by Association Mapping
Source: Front Plant Sci. 2016 Aug 9;7:1188. doi: 10.3389/fpls.2016.01188 (PMC4977947; doi:10.3389/fpls.2016.01188)

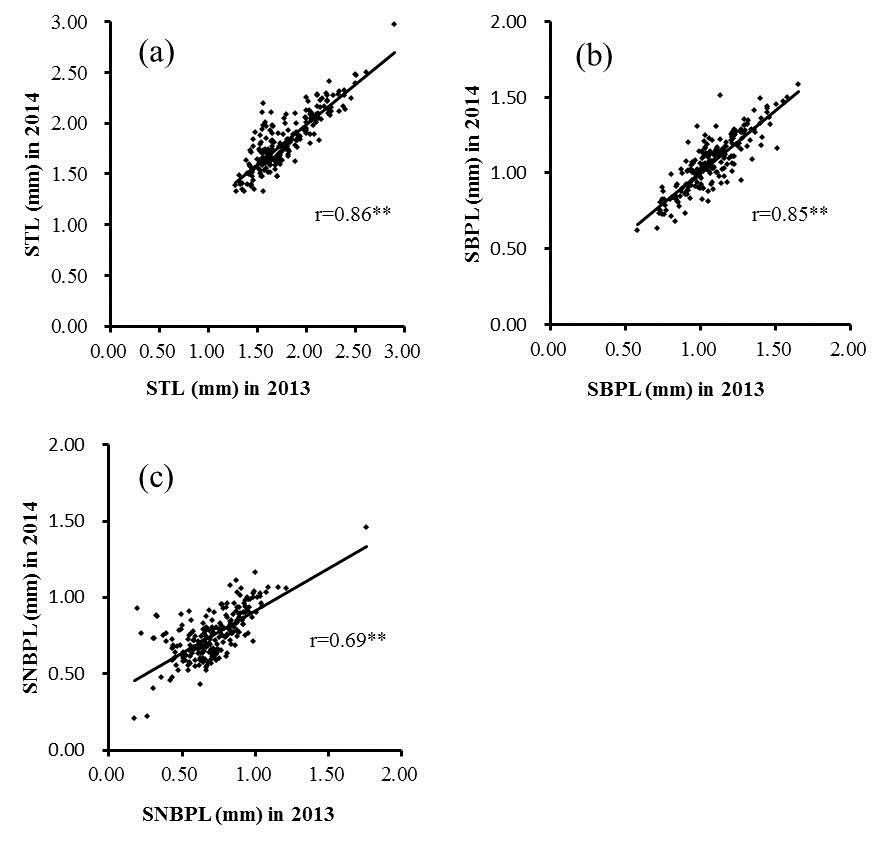

Supplement: Figure S1 — Correlation of stigma traits between 2013 and 2014. A total of 227 rice accessions were grown in the same field in 2013 and 2014 growing season. (A) Correlation of STL between 2013 and 2014. (B) Correlation of SBPL between 2013 and 2014. (C) Correlation of SNBPL between 2013 and 2014. **Significantly correlation at the P < 0.01 (two-tailed). [file Image1.JPEG]

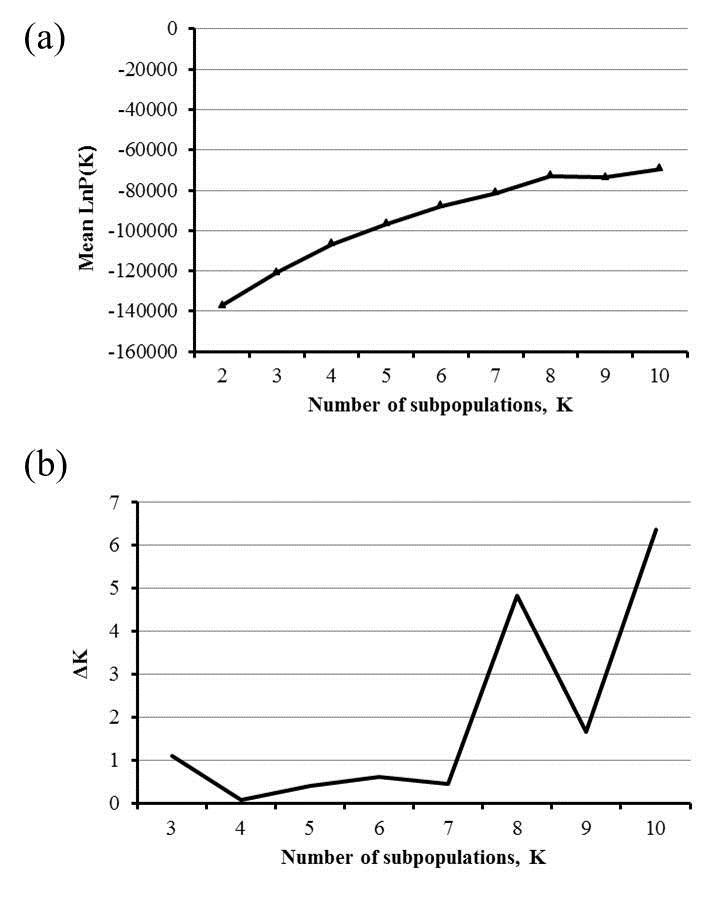

Supplement: Figure S2 — Changes of the mean LnP (K) (A) and ΔK (B). (A) A graph with mean LnP (K) on Y-axis and number of subgroups on X-axis; (B) A graph with ΔK on Y-axis and number of subgroups on X-axis. [file Image2.JPEG]

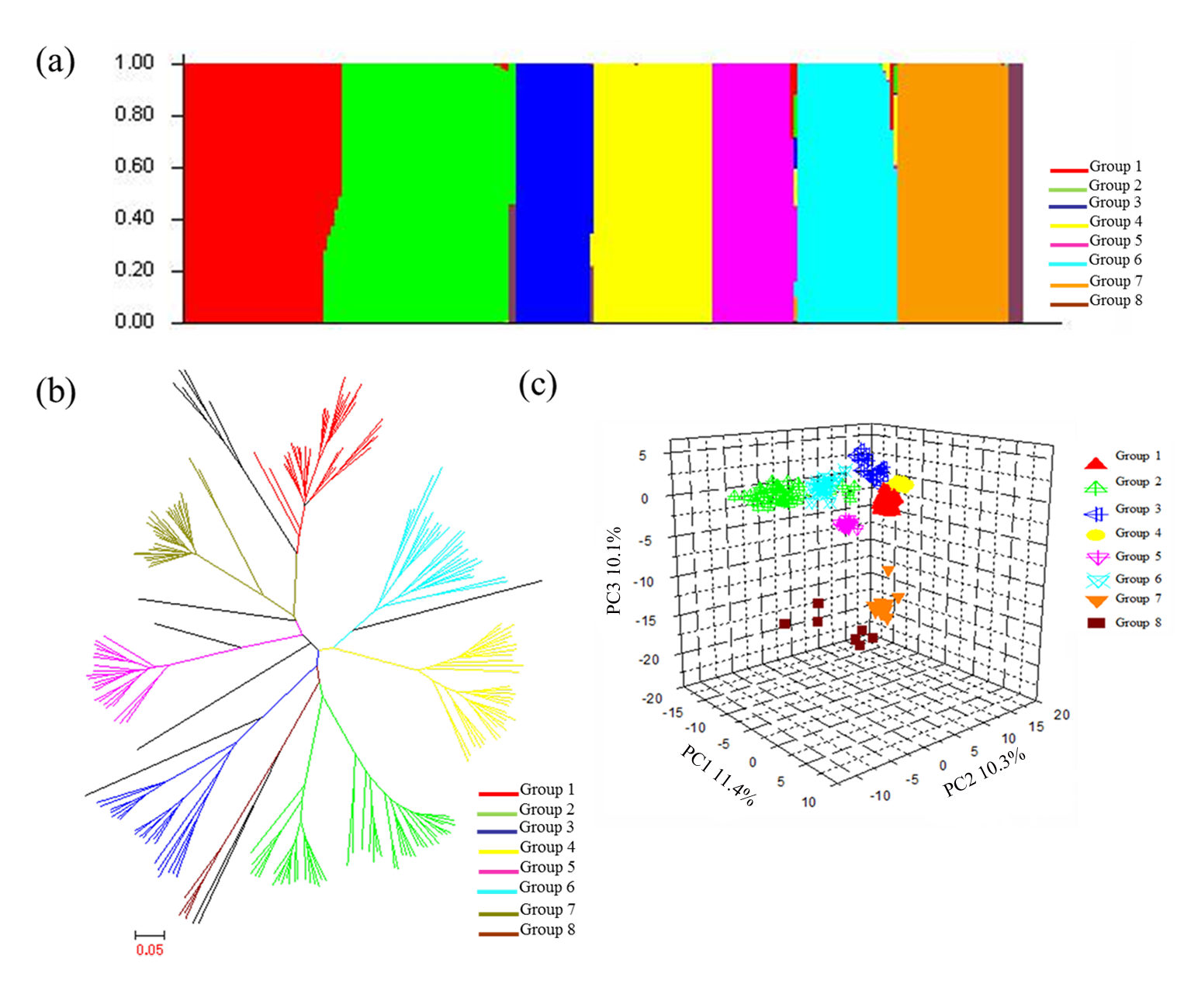

Supplement: Figure S3 — Population structure of 227 Oryza Sativa. (A) STRUCTURE result. The panel is a result of K = 8 (highest likelihood result among replicates). Each accession is represented by a vertical bar. The colored subsections within each vertical bar indicate membership coefficient (Q) of the accession to different clusters. Identified subpopulations are Group1 (red color), Group2 (green color), Group3 (navy blue color), Group4 (yellow color), Group5 (purple color), Group6 (light blue color), Group7 (brown color) and Group8 (caramel color). Accessions marked by group name are considered non-admixed (more than 0.9 assignment to one group in STRUCTURE analysis with K = 8). (B) Result of principal component analysis. Each point corresponds to an individual, and the different color slices correspond to the group assignment matrix (Q matrix) of the STRUCTURE result for K = 8. Numbers in parentheses beside each axis denote the amount of variance explained by that axis. (C) Neighbor-joining tree. The different colored groups approximately correspond to Groups 1–8. Colored branches represent the non-admixed individuals within each corresponding group. Gray branches respent admixed individuals (< 0.9 assignment assigned to any group in STRUCTURE analysis with K = 8). [file Image3.TIF]

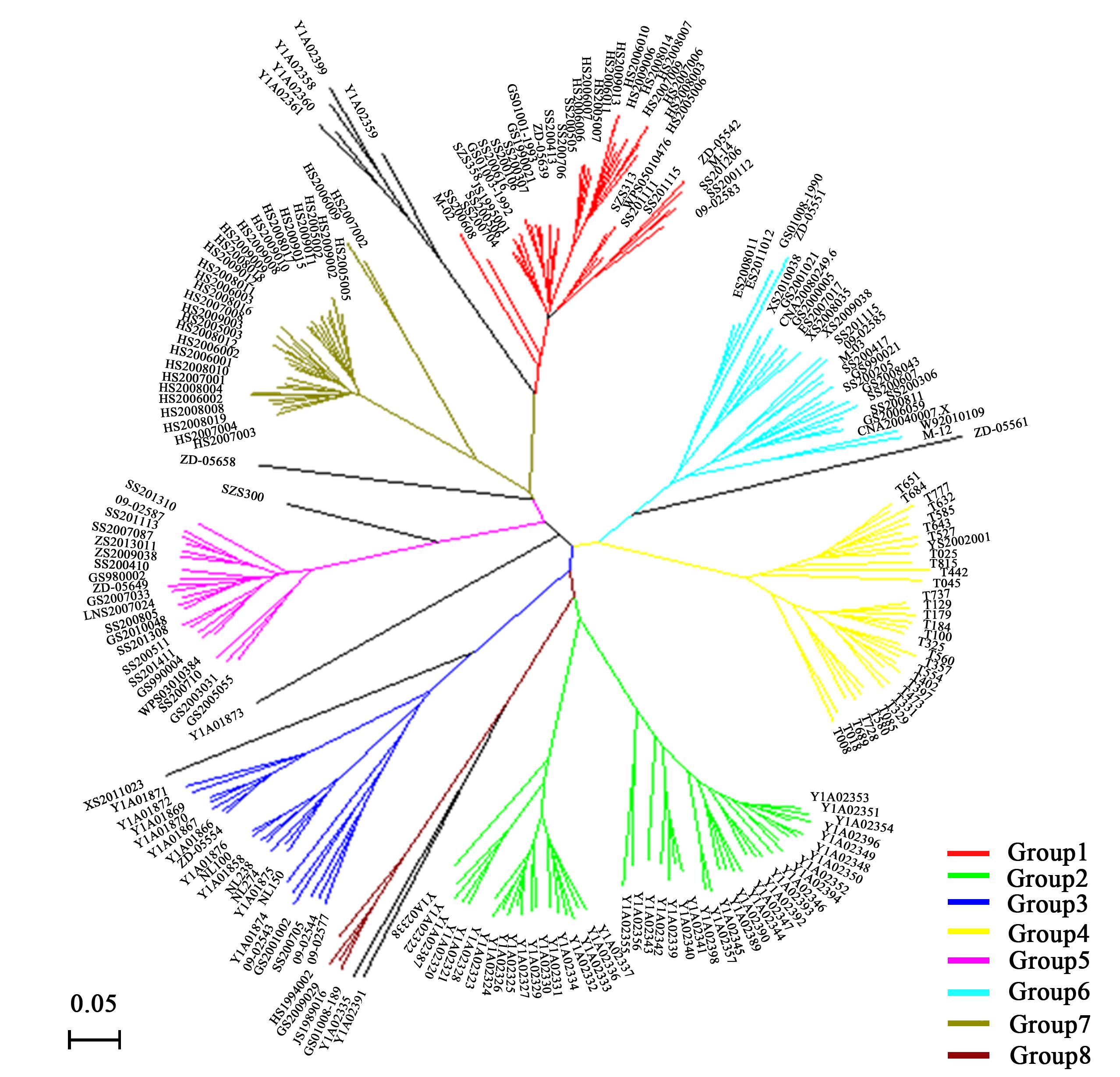

Supplement: Figure S4 — Neighbor-joining tree constructed from Nei's (1983) genetic distance of 249 SSRs. Each branch is corresponding to each accession ID. [file Image4.JPEG]

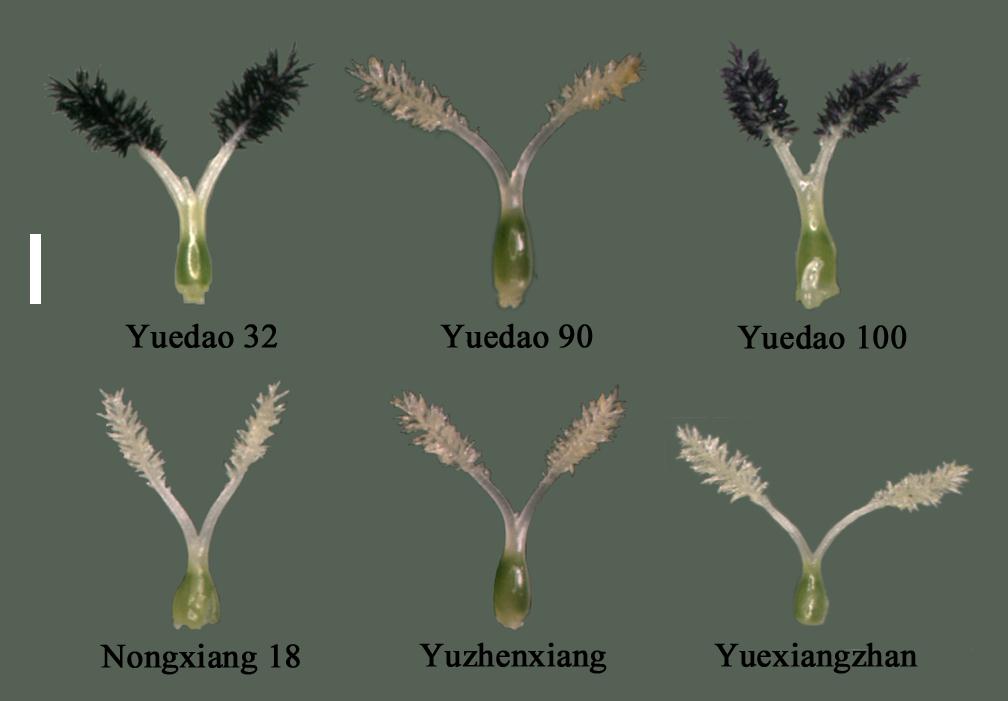

Supplement: Figure S5 — Rice stigma morphology of the 6 elite parents included in the elite crosses predicted. [file Image5.JPEG]
