# Supplementary material for: QTL Detection and Elite Alleles Mining for Stigma Traits in Oryza sativa by Association Mapping
Source: Front Plant Sci. 2016 Aug 9;7:1188. doi: 10.3389/fpls.2016.01188 (PMC4977947; doi:10.3389/fpls.2016.01188)
Supplement: Supp. Table S3 — Locus-by-locus AMOVA for O. sativa based on Structure groups. [file Table3.DOC]

**Table S3** Locus-by-locus AMOVA for *O.* sativa based on Structure groups

| Source of variation | d.f. | Sum of squares | Variance components | Percentage of variation | *P* value |
| --- | --- | --- | --- | --- | --- |
| Among populations | 6 | 17017.64 | 52.25 | 67.88 | <0.0000 |
| Among individuals within populations | 204 | 9090.32 | 24.72 | 32.12 | <0.0000 |
| Total | 210^*^ | 26107.96 | 76.97 |  |  |

* means the total number of nonadmixed individuals of 7 subgroups (>0.9 assignment in Q matrix).
