# Supplementary material for: QTL Detection and Elite Alleles Mining for Stigma Traits in Oryza sativa by Association Mapping
Source: Front Plant Sci. 2016 Aug 9;7:1188. doi: 10.3389/fpls.2016.01188 (PMC4977947; doi:10.3389/fpls.2016.01188)
Supplement: Supp. Table S4 — Positive elite alleles, phenotypic effect value and typical materials for stigma traits and grain length. [file Table4.DOC]

**Table S4** The list for QTLs identified from this study and shared in previous studies

| Trait | SSR  marker | Chromosome | Start position  /bp^a^ | End position  /bp^a^ | QTL reported in the previous studies | | |
| --- | --- | --- | --- | --- | --- | --- | --- |
|  |  |  |  |  | Start position  /bp^a^ | End position /bp^a^ | Reference |
| Stigma length | RM5389 | 1 | 35,732,311 | 35,732,554 | 31,974,410 | 36,352,081 | Uga *et al*., (2010) |
|  | RM450 | 2 | 28,628,206 | 28,628,348 | 28,353,328 | 30,270,847 | Uga *et al*., (2010) |
|  | RM7598 | 2 | 29,821,946 | 29,822,105 | 28,353,328 | 30,270,847 | Uga *et al*., (2010) |
|  | RM280 | 4 | 34,989,558 | 34,989,727 | 20,044,821 | 35,072,135 | Uga *et al*., (2010) |
|  | RM7579 | 6 | 23,501,966 | 23,502,083 | 22,400,122 | 26,729,079 | Uga *et al*., (2003) |
| Stigma brush-shaped part length | RM280 | 4 | 34,989,558 | 34,989,727 | 20,044,821 | 35,072,135 | Uga *et al*., (2010) |
| Stigma non-brush-shaped part length | RM5389 | 1 | 35,732,311 | 35,732,554 | 31,974,410 | 36,352,081 | Uga *et al*., (2010) |
|  | RM450 | 2 | 28,628,206 | 28,628,348 | 28,353,328 | 30,270,847 | Uga *et al*., (2010) |
|  | RM7598 | 2 | 29,821,946 | 29,822,105 | 28,353,328 | 30,270,847 | Uga *et al*., (2010) |
|  | RM280 | 4 | 34,989,558 | 34,989,727 | 20,044,821 | 35,072,135 | Uga *et al*., (2010) |
| Grain length | RM450 | 2 | 28,628,206 | 28,628,348 | 21,580,841 | 35,662,199 | Redoňa & Mackill, (1998) |
|  | RM7598 | 2 | 29,821,946 | 29,822,105 | 21,580,841 | 35,662,199 | Redoňa & Mackill, (1998) |
|  | RM282 | 3 | 12,407,382 | 12,407,510 | 10,572,842 | 21,985,804 | Tan *et al*., (2000) |
|  |  |  |  |  | 8,320,124 | 13,221,665 | Aluko *et al*., (2004) |
|  |  |  |  |  | 9,395,201 | 29,521,508 | Li *et al*., (2004) |
|  |  |  |  |  | 12,407,382 | 24,524,811 | Wang *et al*., (2012) |
|  | RM6712 | 3 | 35,226,547 | 35,226,934 | 33,228,813 | 37,452,807 | Redoňa & Mackill, (1998) |
|  |  |  |  |  | 13,933,574 | 36,249,613 | Bai *et al*., (2010) |
|  | RM6314 | 4 | 18,444,943 | 18,445,111 | 14,706,596 | 19,430,421 | Redoňa & Mackill, (1998) |
|  | RM136 | 6 | 8,751,462 | 8,751,563 | 6,230,045 | 30,864,999 | Dang *et al*., (2015) |
|  | RM2530 | 7 | 15,569,266 | 15,569,409 | 12,544,087 | 24,085,506 | Redoňa & Mackill, (1998) |
|  | RM6976 | 8 | 23,555,534 | 23,555,817 | 21,427,234 | 26,700,842 | Agrama *et al*., (2007) |
|  |  |  |  |  | 23,555,534 | 23,555,817 | Dang *et al*., (2015) |
|  | RM1125 | 10 | 17,771,479 | 17,771,625 | 16,619,676 | 17,845,339 | Redoňa & Mackill, (1998) |
|  |  |  |  |  | 14,156,812 | 22,492,203 | Li *et al*., (2004) |
|  |  |  |  |  | 16,012,317 | 20,913,428 | Wang *et al*., (2011) |

^a^ The estimated physical position (bp) was inferred the Gramene (http://www.gramene.org/markers) and NCBI (http://blast.ncbinlm.nih.gov/Blast.cgi)
