# Supplementary material for: QTL Detection and Elite Alleles Mining for Stigma Traits in Oryza sativa by Association Mapping
Source: Front Plant Sci. 2016 Aug 9;7:1188. doi: 10.3389/fpls.2016.01188 (PMC4977947; doi:10.3389/fpls.2016.01188)
Supplement: Supp. Table S5 — Parental combinations and numbers of elite alleles after combinations predicted from association mapping of stigma traits and grain length. [file Table5.DOC]

**Table S5** Positive elite alleles, phenotypic effect value and typical materials for stigma length traits and grain length

| Trait | Locus-allele  /bp | Phenotypic effect value | | | Typical carrier material |
| --- | --- | --- | --- | --- | --- |
|  |  | 2013 | 2014 | Mean |  |
| STL | RM5389-110 | 0.136 | 0.13 | 0.133 | Kendao 12hao |
|  | RM5389-120 | 0.144 | 0.15 | 0.147 | Yuedao 32 |
|  | RM450-155 | 0.073 | 0.077 | 0.075 | Yuedao 100 |
|  | RM450-135 | 0.228 | 0.243 | 0.235 | Yuedao 32 |
|  | RM7598-95 | 0.131 | 0.124 | 0.127 | Kendao 12hao |
|  | RM7598-115 | 0.142 | 0.151 | 0.146 | Yuedao 100 |
|  | RM280-175 | 0.106 | 0.094 | 0.100 | Yuexiangzhan |
|  | RM7579-90 | 0.129 | 0.118 | 0.123 | Nongxiang 18 |
|  | RM7579-80 | 0.194 | 0.185 | 0.189 | Yuedao 32 |
|  | RM5753-205 | 0.139 | 0.130 | 0.135 | Yuzhenxiang |
|  | RM5753-200 | 0.160 | 0.164 | 0.162 | Yuedao 32 |
|  |  |  |  |  |  |
| SBPL | RM280-175 | 0.072 | 0.073 | 0.072 | Nongxiang 18 |
|  | RM136-110 | 0.038 | 0.035 | 0.037 | Yuedao 90 |
|  | RM136-100 | 0.031 | 0.033 | 0.032 | Songjing 11hao |
|  | RM136-95 | 0.060 | 0.045 | 0.053 | Yuedao 32 |
|  | RM136-200 | 0.110 | 0.112 | 0.111 | Yuexiangzhan |
|  |  |  |  |  |  |
| SNBPL | RM5389-110 | 0.066 | 0.056 | 0.061 | Kendao 12hao |
|  | RM5389-120 | 0.136 | 0.122 | 0.129 | Yuedao 32 |
|  | RM450-155 | 0.036 | 0.025 | 0.031 | Yuedao 100 |
|  | RM450-135 | 0.226 | 0.240 | 0.233 | Yuedao 32 |
|  | RM7598-95 | 0.073 | 0.085 | 0.079 | Kendao 12hao |
|  | RM7598-115 | 0.108 | 0.117 | 0.113 | Yuedao 100 |
|  | RM280-175 | 0.074 | 0.068 | 0.071 | Yuexiangzhan |
|  | RM559-155 | 0.109 | 0.095 | 0.102 | Nongxiang 18 |
|  | RM5753-205 | 0.113 | 0.14 | 0.126 | Yuzhenxiang |
|  | RM5753-200 | 0.154 | 0.164 | 0.159 | Yuedao 32 |
|  | RM6327-200 | 0.096 | 0.088 | 0.092 | Yuedao 32 |
|  | RM6327-180 | 0.117 | 0.125 | 0.121 | Yuedao 90 |
|  |  |  |  |  |  |
| GL | RM128-150 | 0.221 | 0.184 | 0.202 | Yuedao 32 |
|  | RM128-180 | 0.513 | 0.548 | 0.530 | Yuexiangzhan |
|  | RM5389-130 | 0.435 | 0.455 | 0.445 | Nongxiang 18 |
|  | RM5389-120 | 0.770 | 0.742 | 0.756 | Yuedao 32 |
|  | RM450-155 | 0.445 | 0.481 | 0.463 | Yuedao 100 |
|  | RM450-145 | 0.493 | 0.594 | 0.544 | Yuexiangzhan |
|  | RM450-135 | 1.276 | 1.169 | 1.222 | Yuedao 32 |
|  | RM7598-100 | 0.164 | 0.163 | 0.164 | Texianzhan 25 |
|  | RM7598-115 | 1.028 | 0.982 | 1.005 | Yuedao 100 |
|  | RM282-120 | 0.316 | 0.312 | 0.314 | Suijing 12 |
|  | RM282-140 | 0.884 | 0.897 | 0.891 | Texianzhan 25 |
|  | RM282-155 | 1.068 | 1.024 | 1.046 | Yuedao 32 |
|  | RM6712-85 | 0.720 | 0.719 | 0.720 | Yuedao 32 |
|  | RM6712-100 | 0.829 | 0.931 | 0.880 | Nongxiang 18 |
|  | RM6314-175 | 0.366 | 0.382 | 0.374 | Yuexiangzhan |
|  | RM280-175 | 0.143 | 0.138 | 0.141 | Yuzhenxiang |
|  | RM136-110 | 0.543 | 0.550 | 0.546 | Yuedao 89 |
|  | RM136-200 | 1.599 | 1.624 | 1.612 | Yuexiangzhan |
|  | RM2530-145 | 0.175 | 0.166 | 0.171 | Texianzhan 25 |
|  | RM2530-130 | 0.172 | 0.155 | 0.163 | Suijing 12 |
|  | RM2530-160 | 0.919 | 0.881 | 0.900 | Yuedao 32 |
|  | RM6976-265 | 0.469 | 0.416 | 0.442 | 9311 |
|  | RM6976-155 | 0.600 | 0.623 | 0.612 | Yuedao 89 |
|  | RM6976-285 | 0.856 | 0.826 | 0.841 | Nongxiang 21 |
|  | RM6976-135 | 1.493 | 1.291 | 1.392 | Yuedao 32 |
|  | RM1125-145 | 0.171 | 0.155 | 0.163 | Nongxiang 18 |
|  | RM1125-155 | 0.670 | 0.727 | 0.698 | Yuexiangzhan |
|  | RM1125-175 | 1.279 | 1.147 | 1.213 | Yuedao 51 |
|  | RM6327-180 | 0.642 | 0.731 | 0.686 | Yuedao 90 |
|  | RM6327-190 | 0.764 | 0.811 | 0.787 | Yuexiangzhan |
