# Supplementary material for: QTL Detection and Elite Alleles Mining for Stigma Traits in Oryza sativa by Association Mapping
Source: Front Plant Sci. 2016 Aug 9;7:1188. doi: 10.3389/fpls.2016.01188 (PMC4977947; doi:10.3389/fpls.2016.01188)
Supplement: Supp. Table S6 — Elite alleles carried by the superior parents for stigma traits and grain length and corresponding phenotypic effect. [file Table6.DOC]

**Table S6** Parental combinations and numbers of elite alleles after combinations predicted from association mapping of stigma length traits and grain length

| Trait | Parental combination | No. of elite alleles predicted |
| --- | --- | --- |
| STL | Yuedao 32 × Yuedao 100 | 5 |
|  | Yuedao 32 × Nongxiang 18 | 5 |
|  | Yuedao 32 × Yuzhenxiang | 5 |
|  | Yuedao 32 × Yuexiangzhan | 5 |
|  | Yuedao 100 × Yuzhenxiang | 5 |
|  |  |  |
| SBPL | Yuedao 32 × Nongxiang 18 | 2 |
|  | Yuedao 32 × Yuzhanxiang | 2 |
|  | Yuedao 90 × Nongxiang 18 | 2 |
|  | Nongxiang 18 × Yuexiangzhan | 2 |
|  | Yuedao 90 × Yuexiangzhan | 2 |
|  |  |  |
| SNBPL | Yuedao 32 × Yuedao 90 | 6 |
|  | Yuedao 32 × Yuzhenxiang | 6 |
|  | Yuedao 32 × Yuexiangzhan | 6 |
|  | Yuedao 90 × Yuexiangzhan | 7 |
|  | Yuedao 32 × Yuedao 100 | 6 |
|  |  |  |
| GL | Yuedao 51 × Yuzhenxiang | 13 |
|  | Yuedao 51 × Yuexiangzhan | 13 |
|  | Yuedao 32 × Yuexiangzhan | 13 |
|  | Yuedao 90 × Yuexiangzhan | 13 |
|  | Yuedao 32 × Yuzhenxiang | 13 |
