# Supplementary material for: QTL Detection and Elite Alleles Mining for Stigma Traits in Oryza sativa by Association Mapping
Source: Front Plant Sci. 2016 Aug 9;7:1188. doi: 10.3389/fpls.2016.01188 (PMC4977947; doi:10.3389/fpls.2016.01188)
Supplement: Supp. Table S7 — The list for QTLs identified from this study and shared in previous studies. [file Table7.DOC]

**Table S7** Elite alleles carried by the superior parents for stigma length traits and grain length and corresponding phenotypic effect

| Trait | Super parent | Locus-allele (Corresponding phenotypic effect value) |
| --- | --- | --- |
| STL | Yuedao 32 | RM5389-120(0.147), RM450-135 (0.235), RM7579-80 (0.189), RM5753-200 (0.162) |
|  | Yuedao 100 | RM450-155 (0.075), RM7598-115 (0.146), RM7579-90 (0.123) |
|  | Nongxiang 18 | RM280-175 (0.100), RM7579-90 (0.123) |
|  | Yuzhenxiang | RM280-175 (0.100), RM5753-205(0.135) |
|  | Yuexiangzhan | RM280-175 (0.100), RM7579-90 (0.123) |
|  |  |  |
| SBPL | Yuedao 32 | RM136-95 (0.053) |
|  | Yuedao 90 | RM136-110 (0.037) |
|  | Nongxiang 18 | RM280-175 (0.072), RM136-200 (0.111) |
|  | Yuzhenxiang | RM280-175 (0.072), RM136-200 (0.111) |
|  | Yuexiangzhan | RM280-175 (0.072), RM136-100 (0.032) |
|  |  |  |
| SNBPL | Yuedao 32 | RM5389-120 (0.129), RM450-135 (0.233), RM559-155 (0.102), RM5753-200 (0.159), RM6327-200 (0.092) |
|  | Yuedao 90 | RM5389-120 (0.129), RM450-155 (0.031), RM7598-115 (0.113), RM559-155 (0.102), RM5753-200 (0.159), RM6327-180 (0.121) |
|  | Yuedao 100 | RM450-155 (0.031), RM7598-115 (0.113), RM559-155 (0.102) |
|  | Yuzhenxiang | RM280-175 (0.071), RM559-155 (0.102), RM5753-205 (0.126) |
|  | Yuexiangzhan | RM280-175 (0.071), RM559-155 (0.102) |
|  |  |  |
| GL | Yuedao 32 | RM128-150 (0.202), RM5389-120 (0.756), RM282-155 (1.046), RM450-135 (1.222), RM7598-100 (0.164), RM6712-85 (0.720), RM2530-160 (0.900), RM6976-135 (1.392) |
|  | Yuedao 51 | RM128-150 (0.202), RM5389-120 (0.756), RM282-155 (1.046), RM450-135 (1.222), RM7598-100 (0.164), RM6712-85 (0.720), RM2530-160 (0.900), RM6976-135 (1.392), RM1125-175 (1.213) |
|  | Yuedao 90 | RM5389-120 (0.756), RM282-155 (1.046), RM450-155 (0.463), RM7598-115 (1.005), RM6712-85 (0.720), RM6314-175 (0.374), RM136-110 (0.546), RM2530-160 (0.900), RM6976-155 (0.612), RM6327-180 (0.686) |
|  | Yuzhenxiang | RM128-180 (0.530), RM5389-130 (0.445), RM282-140 (0.891), RM450-145 (0.544), RM7598-100 (0.164), RM6712-100 (0.880), RM6314-175 (0.374), RM280-175 (0.141), RM136-200 (1.612), RM6976-285 (0.841), RM1125-145 (0.163), RM6327-190 (0.787) |
|  | Yuexiangzhan | RM128-180 (0.530), RM5389-130 (0.445), RM450-145 (0.544), RM7598-100 (0.164), RM6712-100 (0.880), RM6314-175 (0.374), RM280-175 (0.141), RM136-200 (1.612), RM2530-145 (0.171), RM6976-285 (0.841), RM1125-155 (0.698), RM6327-190 (0.787) |
